# Construction and external validation of a nomogram model for predicting the risk of esophageal stricture after endoscopic submucosal dissection: a multicenter case–control study

**DOI:** 10.1186/s12876-023-02855-8

**Published:** 2023-07-01

**Authors:** Zhao Mu, Xiao Tang, Jingting Wang, Yulin Chen, Kui Cui, Xingyu Rao, Juan Li, Guodong Yang

**Affiliations:** 1 Department of Gastroenterology, The Affiliated Hospital of North Sichuan Medical College, Sichuan, China; 2 Department of Gastroenterology, Sichuan Mianyang 404 Hospital, Mianyang, Sichuan China; 3 Department of Gastroenterology, Langzhong People’s Hospital, Langzhong, Sichuan China; 4 Department of Clinical Medicine, North Sichuan Medical College, Nanchong, Sichuan China

**Keywords:** Endoscopic submucosal dissection, Esophageal stricture, Risk factors, Nomogram, External validation

## Abstract

Esophageal stricture is a common complication after endoscopic submucosal dissection (ESD) for superficial esophageal cancer and precancerous lesions, we intend to investigate the independent risk factors of esophageal stricture after ESD by adding the data of included living habits, established a nomogram model to predict the risk of esophageal stricture, and verified it by external data. The clinical data and living habits of patients with early esophageal cancer and precancerous lesions who underwent ESD in the Affiliated Hospital of North Sichuan Medical College and Langzhong People's Hospital from March 2017 to August 2021 were retrospectively collected. The data collected from the two hospitals were used as the development group (*n* = 256) and the validation group (*n* = 105), respectively. Univariate and multivariate logistic regression analyses were used to determine independent risk factors for esophageal stricture after ESD and establish a nomogram model for the development group. The prediction performance of the nomogram model is internally and externally verified by calculating C-Index and plotting the receiver operating characteristic curve (ROC) and calibration curve, respectively. The results showed that Age, drinking water temperature, neutrophil–lymphocyte ratio, the extent of esophageal mucosal defect, longitudinal diameter of resected mucosa, and depth of tissue invasion (*P* < 0.05) were independent risk factors for esophageal stricture after ESD. The C-Index of the development group and validation group was 0.925 and 0.861, respectively. The ROC curve and area under the curve (AUC) of the two groups suggested that the discrimination and prediction performance of the model were good. The two groups of calibration curves are consistent and almost overlap with the ideal calibration curve, indicating that the predicted results of this model are in good agreement with the actual observed results. In conclusion, this nomogram model has a high accuracy for predicting the risk of esophageal stricture after ESD, providing a theoretical basis for reducing or avoiding esophageal stricture and guiding clinical practice.

**Supplementary Information:**

The online version contains supplementary material available at 10.1186/s12876-023-02855-8.

## Introduction

Esophageal cancer (EC) is the seventh most common cancer and the sixth most common cause of cancer death in the world, and new cases and deaths in China account for 53.7% and 55.7% of the global total, respectively [1]. Esophageal squamous cell carcinoma (ESCC) is the main histological type, the 5-year overall survival rate for patients with advanced ESCC is less than 15%, while in patients diagnosed early in the disease, this may reach 85% [2]. Therefore, early diagnosis and treatment of esophageal cancer can significantly reduce its mortality and is the key to improving patient survival and prognosis.

At present, with the development of endoscopic techniques, endoscopic submucosal dissection (ESD) has gradually become the treatment of choice for early esophageal cancer and precancerous lesions [3]. Esophageal stricture, as one of the major complications after ESD, is characterized by varying degrees of dysphagia [4]. Although it has been shown that lesion size, the extent of mucosal defect, and depth of tissue invasion may be risk factors for esophageal stricture after ESD [5–9]. However, the risk factors of esophageal stricture after ESD are not fully clear, and studies on the effects of living habits on esophageal stricture after ESD have not been reported. If the risk factors of esophageal stricture after ESD can be identified, it will help clinicians to take targeted preventive measures in advance, which is very important to reduce or avoid the occurrence of esophageal stricture.

The nomogram model plays an important role in predicting the individualized assessment of patients, the occurrence of diseases and survival prognosis [10–12]. However, there are few reports on constructing nomogram models to predict esophageal stricture after ESD. Therefore, this paper retrospectively analyzed the relevant data of patients after ESD to identify the independent risk factors of esophageal stenosis after ESD, and then constructed a nomogram model, and assessed the predictive value of this model by means of internal and external validation, providing a theoretical basis for guiding clinical practice.

## Materials and methods

### Patients

In this paper, we retrospectively collected patients with superficial esophageal squamous cell carcinoma and precancerous lesions who underwent esophageal ESD in two hospitals: North Affiliated Hospital of Sichuan Medical College and Langzhong People's Hospital from March 2017 to August 2021, and collected the clinical data and living habits of these patients. The date of first patient data access for this study was January 20, 2022. After strict inclusion criteria and exclusion criteria, 256 patients in the Affiliated Hospital of North Sichuan Medical College and 105 patients in Langzhong People's Hospital were included, and relevant data were collected as the development dataset and validation dataset, respectively. In this study, we routinely used oral steroids in patients with more than 3/4 of the circumferential percentage of mucosal defects, who started oral prednisone acetate at a dose of 30 mg/day on the third postoperative day, which was reduced by 5 mg every 2 weeks. When the dose was reduced to 20 mg/day, 5 mg was reduced every week. Steroids were administered for a total of 8 weeks. In the development group, 67 patients received steroids; in the validation group, 23 patients received steroids. Postoperative re-examinations at endoscopes were performed every 3 months during the first year after ESD to determine whether esophageal strictures developed. This study is a retrospective design and we applied to the ethics committee for a waiver of informed consent, which waived the need for participant consent. The ethical study was performed at The Affiliated Hospital of North Sichuan Medical College under the Declaration of Helsinki and the protocol was approved by the ethics committee of the Affiliated Hospital of North Sichuan Medical College (batch number: 2022ER018-1).

The inclusion criteria included: (1) preoperative comprehensive imaging examination to rule out peripheral lymph nodes and distant metastasis; (2) preoperative endoscopic biopsy findings suggestive of early ESCC or precancerous lesions; (3) endoscopic ultrasonography was performed to rule out cases with tumor invasion depth exceeding the mucosal layer; (4) none of the patients had serious underlying diseases.

Exclusion criteria included: (1) incomplete, blurred, or lost patient-relevant data; (2) Postoperative pathology after ESD revealed esophageal adenocarcinoma, papilloma, spindle cell tumor, etc., with the depth of tissue invasion exceeding 200 μm in the submucosa; (3) loss to follow-up or patient death; (4) patient with a history of other tumors.

Pathological diagnostic criteria: According to the criteria of the World Health Organization [13], pathological diagnosis includes low-grade intraepithelial neoplasia (LGIN), high-grade intraepithelial neoplasia (HGIN), and esophageal squamous cell carcinoma (ESCC).

#### Data collection

In both the development and validation groups, we collected data on the clinical and lifestyle habits of patients with early esophageal cancer and precancerous lesions treated with ESD.

- (1) clinical data: Age, gender, preoperative high neutrophil–lymphocyte ratio (NLR), platelet-lymphocyte ratio (PLR), lympho-monocyte ratio (LMR), the longitudinal length of the resected mucosa, lesion location, en bloc resection, percentage of the circumferential mucosal defect, postoperative pathological type, positive margin, and depth of Invasion.
- (2) living habits data: Long-term smoking, long-term drinking, high protein diet, fruit and vegetable intake, eating speed, pickled food, fried food, and high-temperature water intake.

#### Variables and Definitions:

1. NLR, PLR, and LMR: Preoperative blood routine was collected from patients, including neutrophil, lymphocyte, and monocyte counts. The neutrophil–lymphocyte ratio (NLR), platelet-lymphocyte ratio (PLR), and Lympho-monocyte ratio (LMR) were calculated for each patient, and then the median of the above parameters was found. Those above the median were in the high-value group, and those below the median were in the low-value group.
2. Sites of esophageal lesions: The sites of esophageal lesions were classified according to the latest segmented criteria [14]: 1) cervical esophagus: 15—20 cm from the upper incisors; 2) upper thoracic esophagus: 20—25 cm from the upper incisors; 3) middle thoracic esophagus: 25—30 cm from the upper incisors; 4) lower thoracic esophagus: 30—40 cm from the upper incisors.
3. Depth of tissue invasion: All of them were based on the Paris classification criteria for gastrointestinal tumors in 2002 [15]. The depth of invasion of the diseased tissue was divided into M (confined to the mucosal layer) and SM (infiltrating into the submucosa). Among them, M is divided into M1 (involving the mucosal epithelial layer), M2 (involving the lamina propria), and M3 (involving the Muscularis mucosae); SM is divided into SM1 (depth of invasion no more than 200 μm into the submucosa), SM2 (depth of invasion more than 200 μm into the submucosa), and SM3 (depth of invasion reaching the muscular propria).
4. Assessment of living habits: Referring to the questionnaire survey on living habits by Liu et al. [16]. If the patient usually eats/drinks (e.g., high protein diet, fruit and vegetable intake, pickled food, fried food, high-temperature water intake) more frequently than 3 times a week, it is defined as frequent, and vice versa. Limit the length of time the patient is eating to between 20 and 40 min, defined as fast or slow if the time is below or above this threshold, respectively, and normal otherwise. If the patient's drinking water temperature reaches the threshold of self-perceived warmth, it is defined as high-temperature water intake. Patients who smoked more than 20 cigarettes per day for more than 10 years were defined as long-term smoking; patients who drank more than 100 g per day for more than 10 years were defined as long-term drinking.
5. Esophageal stricture: Esophageal stricture was defined if the patient had symptoms of dysphagia or if standard endoscopy (9.8 mm in diameter) could not pass through the esophagus [17].

### Statistical analysis

Data were statistically analyzed using SPSS 25.0 software and R 4.2.0 software. Enumeration data were compared by chi-square test, measurement data were expressed as mean ± standard deviation $\left(\overline{\mathrm{x}} \pm \mathrm{s }\right)$, and an independent sample t-test was used for comparison. In this study, P-value, odds ratio (OR), and 95% confidence interval (CI) were used to describe the independent risk factors of esophageal stricture after ESD.

SPSS 25.0 software was used for univariate analysis of variables in the developmental group, and multivariate logistic stepwise regression analysis was performed for statistically significant variables (*P* < 0.05) to obtain independent risk factors for esophageal stricture after ESD. Based on the results of multivariate logistic stepwise regression analysis, a nomogram model for predicting esophageal stricture was successfully developed using R 4.2.0 software, and the model fit was assessed by the Hosmer–Lemeshow test.

**Fig. 1:**
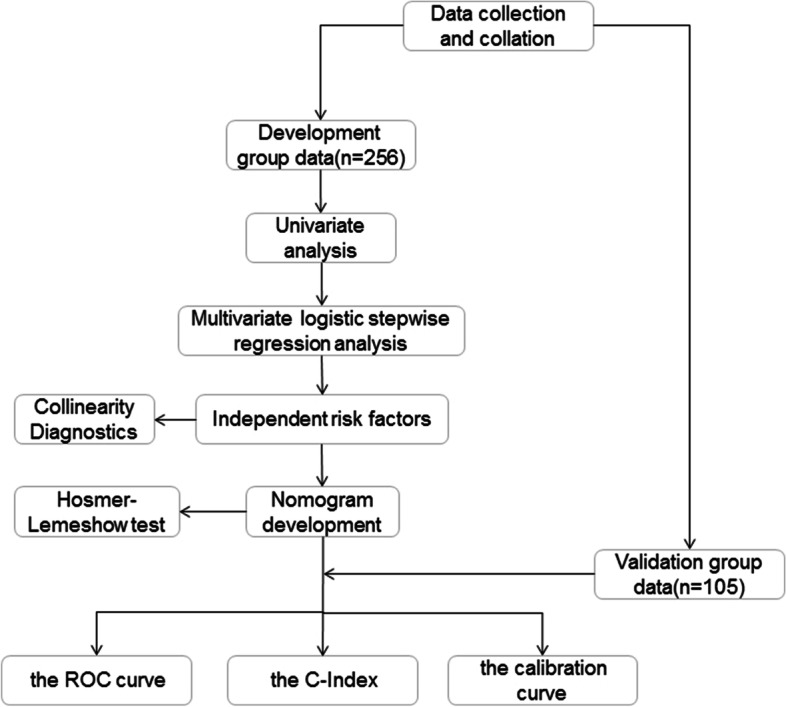
Flowchart for the methods

In this paper, we use development group data for internal validation and validation group data for external validation to test the predictive performance of this nomogram model separately. We use the data of the development group and the validation group to draw the ROC curve and calculate the C-Index, respectively, to evaluate the predictive accuracy of this model, and then use the Bootstrap method to draw the calibration curve of these two groups, and further evaluate whether the prediction probability of this model is consistent with the observation probability. Flowchart for the methods is shown in Fig. 1.

## Results

### Participant characteristics

A total of 361 patients after esophageal ESD were included in this study, consisting of a development group (*n* = 256) and a validation group (*n* = 105), with a ratio of 7:3. In the development group, there were 147 males and 109 females, with an average age of 63.01 ± 8.397 (years); in the validation group, there were 53 males and 52 females, with an average age of 63.10 ± 7.252 (years). All patients were followed up, and postoperative endoscopic reexamination was performed every 3 months in the first year, if there were no significant abnormalities, subsequent reexamination was performed once a year. In the development group, 38 patients developed postoperative stenosis, with an incidence rate of esophageal stenosis of 14.8%; in the validation group, 20 patients developed postoperative stenosis, with an incidence rate of esophageal stenosis of 19.05%. The basic characteristics and living habits of the development group and validation group are shown in Table 1, respectively. There were statistically significant differences between the two groups in terms of BMI, longitudinal diameter of the resected mucosa, en bloc resection, extent of dissected mucosa, long-term smoking, high-protein diet, pickled food, fruit, and vegetable intake, and there was no significant difference in the other variables.

**Table 1:** Baseline characteristics of the development group and validation group

	Development group (*n* = 256)	Validation group (*n* = 105)	t/χ2	*P* Value
Gender (%)			0.002	0.962
Male	147	53		
Female	109	52		
Age (%)			0.002	0.969
> 60	175	72		
≤ 60	81	33		
NLR (%)			0.007	0.935
> 3.186	128	52		
≤ 3.186	128	53		
PLR (%)			0.007	0.935
> 88.57	128	53		
≤ 88.57	128	52		
LMR (%)			0.007	0.935
> 0.2	128	52		
≤ 0.2	128	53		
Longitudinal length, mean ± SD, cm	3.523 ± 1.623	4.657 ± 1.824	5.812	0.000
Lesion location (%)			1.615	0.656
cervical	5	2		
Upper thoracic	45	24		
Middle thoracic	134	54		
Lower thoracic	72	25		
En bloc resection (%)			7.190	0.007
Yes	211	98		
No	45	7		
PCMD (%)			17.644	0.001
< 1/4	32	11		
1/4 ~ 1/2	120	31		
1/2 ~ 3 /4	63	49		
> 3/4	41	14		
Steroid (%)			0.724	0.395
Yes	67	23		
No	189	82		
Postoperative pathological type (%)			4.893	0.087
LGIN	72	18		
HGIN	130	63		
ESCC	54	24		
Positive margin (%)			3.158	0.076
Yes	40	9		
No	216	96		
Depth of Infiltration (%)			0.262	0.609
< M2	199	79		
≥ M2	57	26		

*NLR* Neutrophil-to-lymphocyte ratio, *PLR* Platelet-to-lymphocyte ratio, *LMR* Lympho-monocyte ratio, Longitudinal length Longitudinal length of the resected specimen, *PCMD* Percentage of the circumferential mucosal defect

### Univariate and multivariate logistic regression analysis

Table 2 summarizes the results of developing group univariate and multivariate logistic regression analyses to identify significant predictors of esophageal stricture after ESD. Univariate analysis showed that esophageal stricture after ESD was associated with age, preoperative NLR, longitudinal length of the resected mucosa, PCMD, postoperative pathological type, postoperative hormone use, positive resection margin, depth of invasion, fruit and vegetable intake, eating rate, pickled food, and high-temperature water intake (*P* < 0.05). In order to control for confounding factors, we performed multivariate logistic stepwise regression for the above statistically significant factors. The results showed that age of more than 60 years, long-term high-temperature water intake, preoperative high NLR, esophageal mucosal defect of more than 3/4, longitudinal diameter of resected mucosa, and depth of tissue invasion of more than M2 were independent risk factors for the esophageal stenosis after ESD. We found a cutoff value of 4 cm for the longitudinal length of the resected mucosa by SPSS software, with a Youden index of 0.603, corresponding to a sensitivity of 84.2% and a specificity of 76.1%.

**Table 2:** Univariate and multivariate logistic regression models in the development group

	Univariate analysis		Multivariate analysis	
	OR (95%CI)	P value	OR (95%CI)	P value
Gender	1.256(0.629–2.508)	0.518		
Age	4.642(1.588–13.568)	0.005	11.229(2.315—54.466)	0.003
Hypertension	1.181(0.482–2.893)	0.717		
Diabetes	1.045(0.222–4.914)	0.955		
BMI	1.029(0.913–1.161)	0.637		
NLR	2.147(1.044–4.416)	0.038	5.65(1.782—17.917)	0.003
PLR	1.111(0.557–2.215)	0.765		
LMR	0.606(0.300–1.224)	0.162		
Longitudinal length	1.775(1.405–2.241)	0.000	1.422(1.036—1.951)	0.029
Lesion location	NA	0.751		
En bloc resection	0.935(0.383–2.281)	0.882		
PCMD	NA	0.000	8.564(2.587—28.355)	< 0.001
Steroid	6.064(2.923–12.579)	0.000		
Postoperative pathological type	NA	0.000		
Positive margin	3.132(1.420–6.906)	0.005		
Depth of Infiltration	NA	0.000	4.433(1.602—12.272)	0.004
High protein diet	0.814(0.399–1.659)	0.571		
Fruit and vegetable intake	0.281(0.135–0.586)	0.001		
Eating speed	NA	0.051		
Pickled food	6.131(2.425–15.499)	0.000		
Fried food	4.157(1.115–15.498)	0.034		
High temperature water intake	8.826(3.827–20.354)	0.000	13.378(3.987—44.882)	< 0.001
Long-term smoking	1.026(0.502–2.097)	0.943		
Long-term drinking	0.523(0.194–1.410)	0.200		

*OR* Odds ratio, *CI* Confidence interval;

Variables that are not statistically significant will be excluded in univariate or multivariate analyses

### Nomogram development

**Fig. 2:**
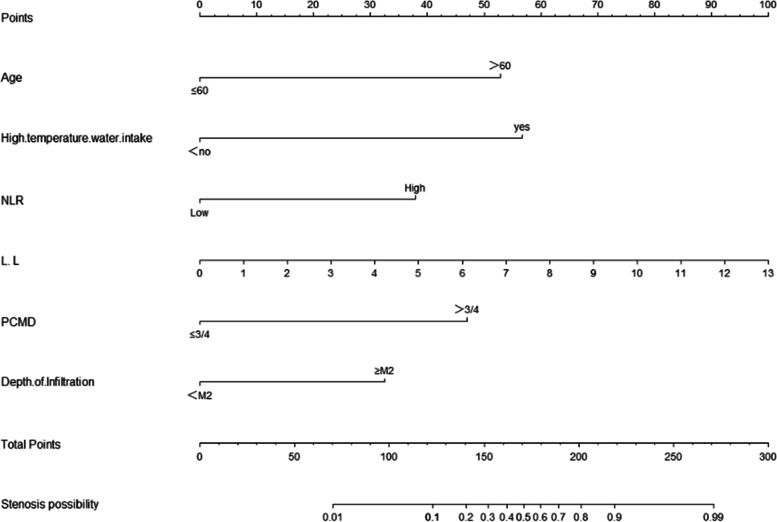
Nomograms for the individualized prediction of esophageal stricture after endoscopic submucosal dissection

Collinearity analysis of age, drinking temperature, NLR, longitudinal diameter of resected mucosa, circumferential range of mucosal defects, and depth of tissue invasion resulted in variance inflation factors (VIFs) of 1.023, 1.034, 1.010, 1.466, 1.496, and 1.094, respectively, which indicated that there was no multicollinearity among the six independent risk factors. Based on the results of logistic regression analysis, we developed an individualized prediction model for esophageal stricture after ESD using R, the nomogram model (Fig. 2). The total risk score is determined by obtaining the point of each relevant factor on the nomogram, adding the scores of all variables, and directly obtaining the risk probability of developing esophageal stricture, if the risk probability is greater than 50%, the patient is predicted to develop esophageal stricture.

### Nomogram validation

**Fig. 3:**
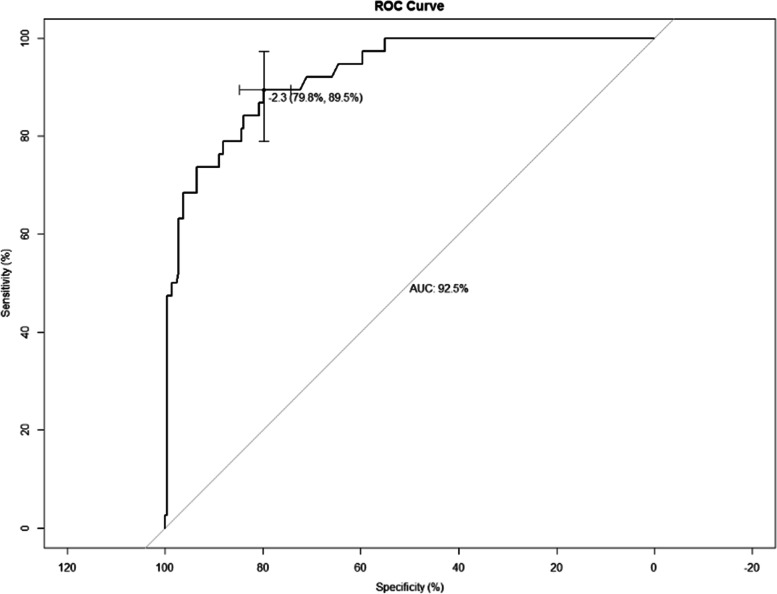
ROC curves for validating the discrimination power of the nomogram in the development group

**Fig. 4:**
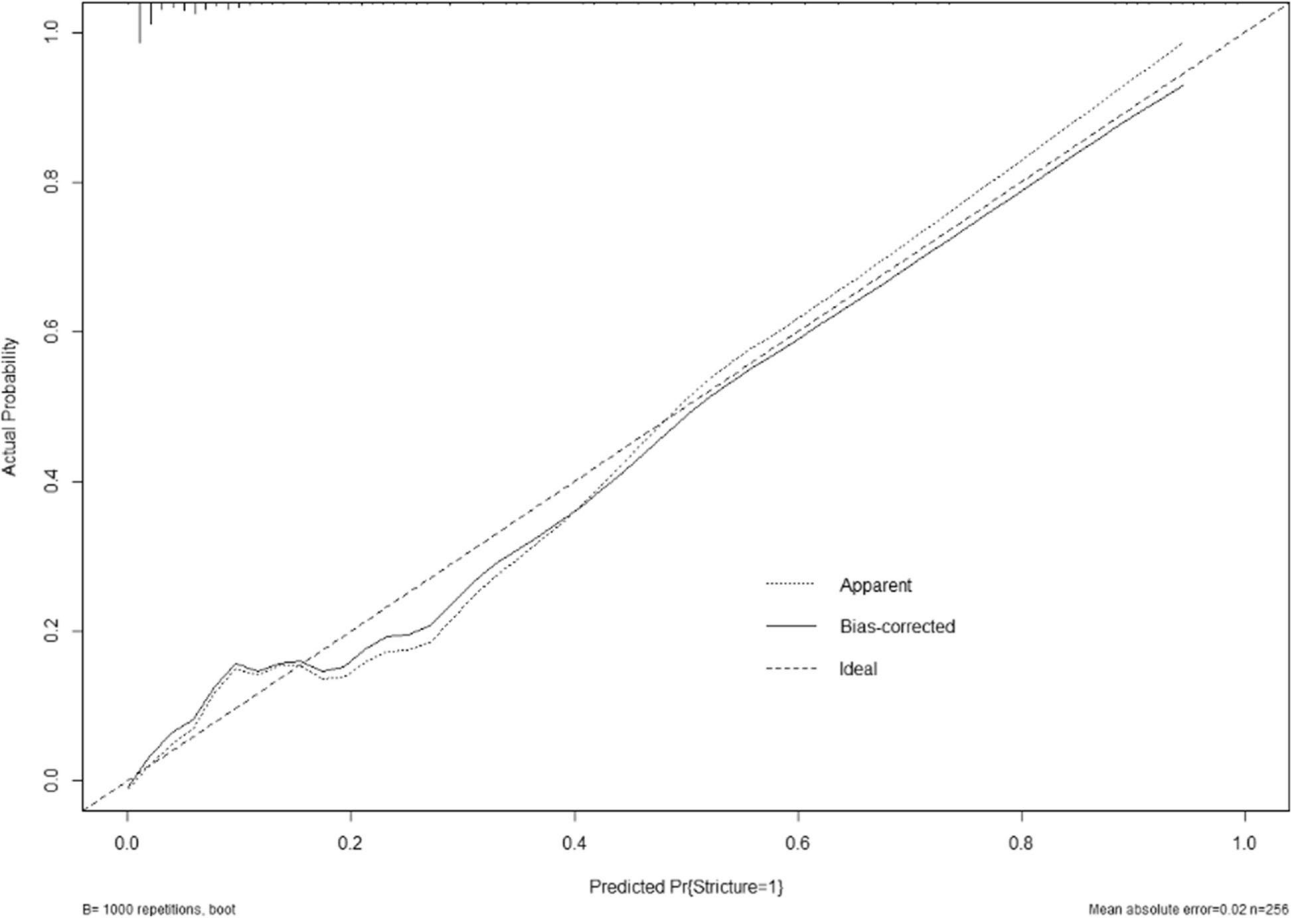
The calibration curve for the test accuracy of the nomogram in the development group

**Fig. 5:**
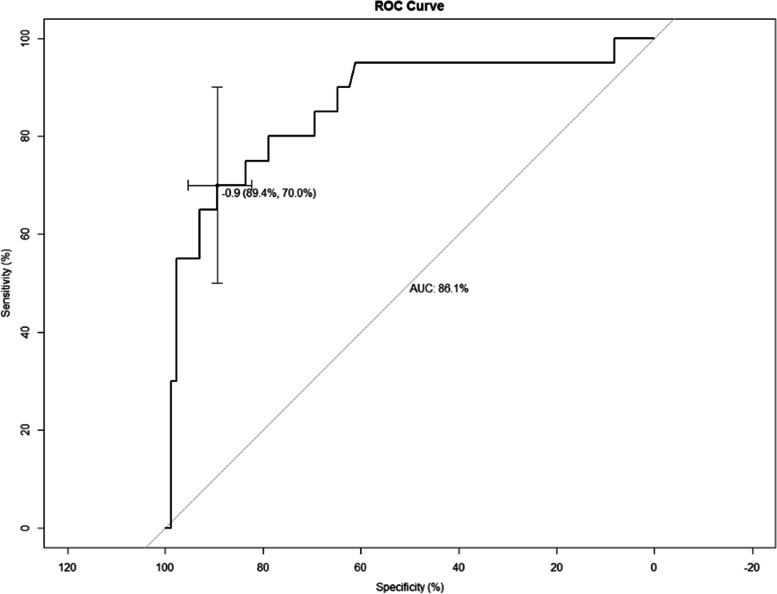
ROC curves for validating the discrimination power of the nomogram in the validation group

**Fig. 6:**
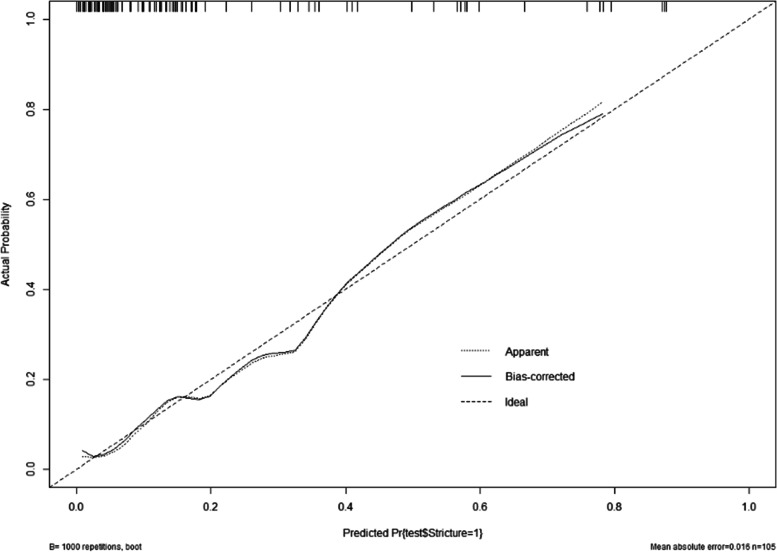
The calibration curve for the test accuracy of the nomogram in the validation group

The fitness of the model was analyzed using the Hosmer–Lemeshow test, and the results showed that the fitness of the model was good and could be used to predict the risk of occurrence (*P* = 0.979). The performance of the nomogram model was verified by calculating the C-Index and plotting the ROC curve and the calibration curve. First, we first internally validate the model. We calculated the C-Index of the development group to be 0.925, plotted the ROC curve (Fig. 3), and showed that the AUC was 92.5%, and the sensitivity and specificity were 89.5% and 79.8%, respectively, indicating that the discrimination and prediction performance of this model was good. The calibration curve (Fig. 4) shown that the apparent appearance of the developed calibration curve almost overlaps with the ideal nomogram calibration curve. We then externally validated the model. We plotted ROC curves using the validation set (Fig. 5), which showed that the AUC was 86.1%, and sensitivity and specificity of 89.4% and 70.0%, respectively, indicating that the model has good predictive performance in different populations. The calibration curve of the validation group (Fig. 6) showed that it was basically consistent with the calibration curve of the development group, indicating that the prediction of the nomogram model was in good agreement with the observation results.

## Discussion

In recent years, ESD has gradually become the treatment strategy of choice for superficial esophageal squamous cell carcinoma and precancerous lesions [4]. However, esophageal stricture after ESD severely affects patients' quality of life. At present, the risk factors of esophageal stricture after ESD are still unclear, the effect of patients' living habits on esophageal stricture after ESD has not been reported, and the prediction model of esophageal stricture after ESD lacks effective external validation. Therefore, this study retrospectively collected the basic data and living habits of patients, used univariate and multivariate analysis to determine the risk factors of esophageal stenosis after ESD, and then established a nomogram model to predict the risk of esophageal stenosis, and validated the model by internal and external validation.

This study found that age of more than 60 years, long-term high-temperature water intake, preoperative high NLR, esophageal mucosal defect of more than 3/4, longitudinal diameter of resected mucosa, and depth of tissue invasion of more than M2 were independent risk factors for esophageal stenosis after ESD (*P* < 0.05). Previous studies have shown that the extent of esophageal mucosal defects, the depth of tissue invasion, and the length of longitudinal mucosal defects are reliable independent risk factors for esophageal stricture after ESD [7, 8, 18–21], but the extent of specific mucosal defects and the length of longitudinal mucosal defects still vary individually in different research reports. Katada et al. found that the frequency of esophageal strictures with mucosal defect circumference above 3/4 and longitudinal diameter > 30 mm was significantly higher in patients [18], thus confirming a correlation between the extent and size of mucosal resection and esophageal strictures. Tang et al. in investigating the risk factors for refractory stricture after extensive ESD, found that the length of longitudinal resection > 50 mm and the depth of tissue invasion above M2 were independent risk factors for refractory esophageal stricture [22]. Ono et al. used stepwise logistic regression analysis to investigate predictors of stricture after ESD and found that circumferential resection > 3/4, longitudinal diameter > 30 mm, and histological depth of M2 were reliable predictors of postoperative esophageal stricture [8]. The results of this study are basically consistent with the conclusions of previous literature.

In previous studies, inflammatory markers such as NLR, PLR, and LMR are often used to predict the stage, differentiation, invasion, and prognosis of tumors, and have good predictive value [23, 24]. The above indicators were included in this study. Statistical analysis showed that preoperative high NLR was an independent risk factor for esophageal stenosis after ESD, and preoperative high NLR was more likely to have postoperative esophageal stenosis (*P* = 0.003, OR = 5.65, 95% CI: 1.782—17.917). In fact, the formation of esophageal stricture itself requires the participation of inflammatory mediators, which play an important role in scar repair [25]. Therefore, inflammatory markers may be one of the risk factors for esophageal stricture.

The incidence of cancer increases with age, which is a risk factor for esophageal cancer and is commonly used to predict the occurrence and prognosis of cancer [26, 27]. The effect of age on esophageal stricture was rarely reported in previous studies, but the results of this study indicated that an age older than 60 years was an independent risk factor for esophageal stricture (*P* = 0.003, OR = 11.229, 95% CI: 2.315—54.466). Funakawa et al. found that there was no significant difference in age, gender, alcohol consumption and smoking, and tumor location between the esophageal stenosis group and the non-stenosis group after ESD [28]. However, this study obtained that age was an independent risk factor for esophageal stricture based on excluding confounding factors, which had some accuracy and reliability. The increased risk of esophageal stricture in elderly patients probably is caused by factors such as trace elements and vitamin deficiency related to the mucosal repair and poor local blood circulation [29–31].

Liu et al. established an age-stratified risk prediction model for esophageal squamous cell carcinoma and its precancerous lesions on the basis of a population-based large endoscopic screening trial, which included predictors of lifestyle habits and dietary status [16]. In recent years, no report has been found that the patient's living habits are included in the risk factors and prediction model of esophageal stenosis. This study, the living habits of patients were included in this study for the first time, and the results of multivariate logistic stepwise regression revealed that long-term high-temperature water intake was an independent risk factor for esophageal stenosis after ESD (*P* < 0.001, OR = 13.378, 95% CI: 3.987—44.882), but not with high-protein diet, fruit and vegetable intake, pickled food, fried food, eating speed, long-term smoking and alcohol consumption (*P* > 0.05). High temperature have been reported to lead to esophageal mucosal edema and inflammation formation, long-term will lead to surrounding fibrous hyperplasia, collagen accumulation, scar contracture, and ultimately esophageal scar stenosis [32]. Therefore, high-temperature water intake probably is one of the causes of esophageal stricture after ESD. However, its accuracy and effectiveness need to be verified by more clinical studies in the future.

In this study, nomogram models were established to predict the risk of esophageal stricture after ESD, and the models were internally and externally validated by calculating C-Index, plotting ROC curves, and calibration curves. The c-index of the development group and the validation group were 0.925 and 0.861, respectively, and the ROC curves of the two groups suggested that the discrimination and prediction performance of the model were good. The two groups of calibration curves were basically consistent and almost overlapped with the ideal calibration curve, indicating that the predictions of the nomogram model were in good agreement with the observations. Since the data sources between the development group and validation group are different, there are some differences in the patient's living habits, physician technical level, etc., but this does not significantly affect the discrimination and calibration of this prediction model. External validation showed that the model had good performance in predicting esophageal stricture after ESD, indicating that the model can be well-suitable for different populations and medical centers, which is important to reduce or avoid the occurrence of esophageal stricture.

However, this study still has some limitations. This study was a multi-center, retrospective, small-sample design, so the clinical effectiveness needed to be verified in a large-sample, prospective study; secondly, the evaluation of the patient's living habits and other indicators had certain subjectivity, and the relevant factors should be quantified by the standard for accurate analysis; lastly, due to the limited factors included in this study, such as the technical level of endoscopists, patient's work, living environment, and other factors may also affect the study outcome, it needs to be considered in the future relevant studies.

## Conclusion

In this study, a nomogram model was established to predict the risk of esophageal stenosis after ESD. Both internal and external validations showed that the model has good predictive performance and can be well adapted to different populations and medical centers. It is a free tool beneficial to clinical risk assessment, which has a positive significance for preventing esophageal stenosis and guiding clinical practice. However, more prospective validation is needed in the future.

## Supplementary Information

**Additional file 1.**

**Additional file 2.**

## Data Availability

The datasets generated and analyzed during the current study are not publicly available for the sake of considering the principle of protecting patient privacy but are available from the respective authors upon reasonable request.

## Abbreviations

ESCC: Esophageal squamous cell carcinoma
ESD: Endoscopic submucosal dissection
LGIN: Low-grade intraepithelial neoplasia
HGIN: High-grade intraepithelial neoplasia
NLR: Neutrophil–lymphocyte ratio
PLR: Platelet-lymphocyte ratio
LMR: Lympho-monocyte ratio
VIFs: Variance Inflation Factors
OR: Odds ratio
95%CI: 95% Credibility Interval
ROC: Receiver Operating Characteristic
AUC: Area Under ROC Curve
BMI: Body mass index
L-D: Longitudinal diameter of the resected specimen
PCMD: Percentage of the circumferential mucosal defect
